# Folic acid: a potential inhibitor against SARS-CoV-2 nucleocapsid protein

**DOI:** 10.1080/13880209.2022.2063341

**Published:** 2022-05-20

**Authors:** Yu-meng Chen, Jin-lai Wei, Rui-si Qin, Jin-ping Hou, Guang-chao Zang, Guang-yuan Zhang, Ting-ting Chen

**Affiliations:** a Laboratory of Tissue and Cell Biology, Lab Teaching & Management Center, Chongqing Medical University, Chongqing, PR China; b Department of Gastrointestinal Surgery, The First Affiliated Hospital of Chongqing Medical University, Chongqing, PR China; c General Surgery of Neonatal Surgery, Liangjiang District, Children's Hospital of Chongqing Medical University, Chongqing, PR China; d Pathogen Biology and Immunology Laboratory, Lab Teaching & Management Center, Chongqing Medical University, Chongqing, PR China

**Keywords:** COVID-19, Chinese medicine, network pharmacology, antivirus

## Abstract

**Context:**

Coronavirus disease 2019 is a global pandemic. Studies suggest that folic acid has antiviral effects. Molecular docking shown that folic acid can act on SARS-CoV-2 Nucleocapsid Phosphoprotein (SARS-CoV-2 N).

**Objective:**

To identify novel molecular therapeutic targets for SARS-CoV-2.

**Materials and methods:**

Traditional Chinese medicine targets and virus-related genes were identified with network pharmacology and big data analysis. Folic acid was singled out by molecular docking, and its potential target SARS-CoV-2 N was identified. Inhibition of SARS-CoV-2 N of folic acid was verified at the cellular level.

**Results:**

In total, 8355 drug targets were potentially involved in the inhibition of SARS-CoV-2. 113 hub genes were screened by further association analysis between targets and virus-related genes. The hub genes related compounds were analysed and folic acid was screened as a potential new drug. Moreover, molecular docking showed folic acid could target on SARS-CoV-2 N which inhibits host RNA interference (RNAi). Therefore, this study was based on RNAi to verify whether folic acid antagonises SARS-CoV-2 N. Cell-based experiments shown that RNAi decreased *mCherry* expression by 81.7% (*p* < 0.001). This effect was decreased by 8.0% in the presence of SARS-CoV-2 N, indicating that SARS-CoV-2 N inhibits RNAi. With increasing of folic acid concentration, *mCherry* expression decreased, indicating that folic acid antagonises the regulatory effect of SARS-CoV-2 N on host RNAi.

**Discussion and conclusions:**

Folic acid may be an antagonist of SARS-CoV-2 N, but its effect on viruses unclear. In future, the mechanisms of action of folic acid against SARS-CoV-2 N should be studied.

## Introduction

The recently discovered severe acute respiratory syndrome coronavirus 2 (SARS-CoV-2) is the causative agent of coronavirus disease 2019 (COVID-19), a respiratory disease that rapidly caused a global pandemic and social and economic disruption (Wu et al. 2020; Zheng et al. 2020). As of September 2, 2021, over 218 million cases of COVID-19 have been reported, and more than 4.52 million lives have been lost globally (https://covid19.who.int). SARS-CoV-2 is a positive-sense, single genomic RNA virus, approximately 30 kb in length, encoding four structural proteins: spike (S), membrane (M), envelope (E), and nucleocapsid (N) proteins (Kim et al. 2020). These structural proteins are components of the mature virus, and play a crucial role in viral structural integrity, or as in the case of the S protein, in host entry (Luan et al. 2020; Wrapp et al. 2020). The S protein binds to the angiotensin-converting enzyme (ACE) receptor on the cytoplasmic membrane of type 2 lung cells and intestinal epithelium. After binding, the S protein is cleaved by the host membrane serine protease TMPRSS2, which promotes virus entry (Sekimukai et al. 2020). The SARS-CoV-2 genome contains 14 open reading frames, preceded by transcriptional regulatory sequences (Wu et al. 2020). The structural and accessory proteins are translated from a set of nested subgenomic (sg) RNAs. Understanding how the virus hijacks the host during infection has promoted the development, characterisation, and deployment of an effective vaccine, antibody prophylaxis, and treatment against SARS-CoV-2, in order to prevent morbidity and mortality, as well as curtail its epidemic spread (Huang et al. 2020; Li et al. 2021; Singh et al. 2021; Zhu 2021).

Relevant Traditional Chinese medicine (TCM) formulations were originally developed for early viral diseases and have been used in the COVID-19 treatment (Akalin et al. 2020). TCM has been extensively used to control the epidemic in China (Girija and Sivan 2022). Shufeng Jiedu and Lianhua Qingwen capsules have positive clinical effects on COVID-19 patients (Liu et al. 2020). Further studies focussing on TCM may lead to the identification of novel antiviral compounds for treating SARS-CoV-2 and other emerging viral diseases (Akalin et al. 2020; National Health Commission 2020). Antiviral medicines such as teicoplanin, arbidol, and chloroquine and its derivatives target mainly the host protease or other cellular proteins, whereas lopinavir/ritonavir, remdesivir, favipiravir, and ribavirin target viral proteins; niclosamide targets both categories (Li et al. 2020). The combination of TCM and conventional Western medicine (CWM) can effectively inhibit SARS-CoV-2 replication and prevent the production of proinflammatory cytokines induced by SARS-CoV-2 infection. Numerous studies have only analysed the therapeutic mechanism of CWM or TCM individually in COVID-19. Therefore, this study integrated these two modes of treatment to explore their generality and characteristics. Based on the ‘disease-target-compound-drug’ concept, network pharmacology systematically analyzes the mechanism of drug action and interaction from multiple levels and angles (Iorio et al. 2010; Jia et al. 2016).

Network pharmacology combines information on the genome, proteome, drugs, diseases, and other related databases, with experimental verification data through the method of big data mining, so as to establish an interrelated disease-target-compound-drug network (Zhang et al. 2018; Alaimo and Pulvirenti 2019). Therefore, this method can be used to systematically analyse the intervention and influence of TCM and CWM on COVID-19, reveal the mechanism of drug action in the human body, correlate the pharmacophores and interactions of various drugs, and screen the core targets. Moreover, the accumulation of big data resources is conducive to screening molecular markers for disease prevention (Lin et al. 2019). Bimolecular targets are the basis of accurate diagnosis and personalised drug design, which can be used to capture specific disease signals in the early stages to formulate appropriate treatment plans (Lin et al. 2019). Molecular target screening for COVID-19 therapy is of great significance for understanding the mechanism of interaction between SARS-CoV-2 and the host. Therefore, it is essential to integrate network pharmacology and Gene Expression Omnibus (GEO) databases to screen COVID-19 molecular therapeutic targets with clinical value. Identifying the regulatory effects of core hub genes based on big data can provide potential targets and original ideas for COVID-19 gene therapy and new drug development.

To this end, in the present study, network pharmacology and GEO databases were integrated to mine and identify COVID-19 molecular therapeutic targets, providing potential targets and ideas for COVID-19 gene therapy and new drug development. The study involved: 1) using the Traditional Chinese Medicines for Systems Pharmacology Database and Analysis Platform (TCMSP), Therapeutic Target Database (TTD), PubChem, and the Comparative Toxicogenomics Database (CTD) to analyse drug interactions and associated phenotypes for SARS-CoV-2 and correlate drug and disease interaction mechanisms to screen key drug targets; 2) using the GEO database to correlate differential genes and drug targets to screen potential antiviral gene therapy targets and construct regulatory networks and key points of SARS-CoV-2 therapeutic drugs; and 3) identifying the regulatory effects of different concentrations of folic acid on the N protein based on the cellular shRNA interference model. The pivotal gene identification provides a target for COVID-19 molecular therapy, as well as ideas for analysing the interactions between the virus and the host.

## Materials and methods

### Active compounds and drug target genes

Chemical ingredients and target genes were collected from the TCMSP (http://tcmspw.com/tcmsp.php). In this database, the final candidate active ingredients were screened based on oral bioavailability ≥30%, drug-likeness ≥0.18, and drug half-life ≥10. The compound target genes were imported into UniProt (https://www.uniprot.org/) to define the species as human, and gene names corresponding to the target genes were obtained. MolAICal software, developed by Bai et al. (2020) at Lanzhou University, was used to calculate the molecular synthetic accessibility score (SAS). The SAS ranges from 0-100, and the larger the value, the easier the synthesis of the compound.

The COVID-19 treatment plan (sixth trial edition) was selected (National Health Commission 2020). The Chinese medicines used are listed in Table 1.

**Table 1.** Chinese medicine in the COVID-19 treatment plan (sixth trial edition).

Latin name	Chinese Pinyin name	Latin name	Chinese Pinyin name
*Ephedra sinica* Stapf	Mahuang	*Amygdalus communis* Vas	Kuxingren
*Cinnanmomi cassia Presl* Cortex	Rougui	*Glycyrrhiza uralensis* Fisch.	Gancao
*Alisma orientalis* (Sam.) Juzep.	Zexie	*Pogostemon cablin* (Blanco) Benth.	Huoxiang
*Polyporus umbellatu* (Pers.) Fr.	Zhuling	*Magnolia officinalis* Rehd et Wils.	Houpu
*Atractylodes macrocephala* Koidz.	Baizhu	*Atractylodes lancea* (Thunb.) DC.	Cangshu
*Pinellia ternate* (Thunb.) Breit.	Banxia	*Amomum tsaoko* Crevostet et Lemarie	Caoguo
*Aster tataricus* L. f.	Ziwan	*Rheum palmatum* L．	Shengdahuang
*Tussilago farfara* L. Sp. Pl.	Donghua	*Anemarrhena asphodeloides* Bunge	Zhimu
*Belamcanda chinensis* (L.) Redouté	Shegan	*Paeonia lactiflora* Pall.	Chishao
*Asarum heterotropoides* Fr. Schmidt	Xixin	*Scrophularia ningpoensis* Hemsl.	Xuansen
*Dioscorea polystachya* Turczaninow	Shanyao	*Forsythia suspensa*	Lianqiao
*Citrus aurantium* L.	Zhishi	*Paeonia suffruticosa* Andr.	Danpi
*Cyrtomium fortunei* J. Sm.	Guanzong	*Coptis chinensis* Franch.	Huanglian
*Cynanchum paniculatum* (Bunge) Kitagawa	Xuchangqing	*Draba nemorosa* L.	Tinglizi
*Eupatorium fortunei* Turcz	Peilan	*Panax ginseng* C. A. Meyer	Rensen
*Scutellaria baicalensis* Georgi	Huangqin	*Cornus officinalis* Sieb. et Zucc.	Shanzhuyu
*Bupleuri chinense*	Chaihu	*Arum ternatum* Thunb.	Fabanxia
*Artemisia annua* L.	Qinghao	*Citrus reticulata*	Chenpi
*Isatis indigotica* Fortune	Daqingye	*Codonopsis pilosula* (Franch.) Nannf.	Dangsen
*Reynoutria japonica* Houtt.	Huzhang	*Glehnia littoralis* Fr. Schmidt ex Miq.	Beishasen
*Verbena officinalis* L.	Mabiancao	*Schisandra chinensis* Fructus	Wuweizi
*Phragmites australis* (Cav.) Trin. ex Steud.	Lugen	*Morus alba* L.	Sangye
*Citrus grandis* (L.) Osbeck	Huajuhong	*Areca catechu* L.	Binglang
*Notopterygium incisum* Ting ex H. T. Chang	Qianghuo	*Salvia miltiorrhiza* Bge.	Dansen

### Differentially expressed genes induced by SARS-CoV-2 infected NHEB and A549 cell lines

The differentially expressed genes (DEGs) between EGFR-TKI sensitive and resistant genes were obtained from the public GEO database. The GSE147507 dataset includes SARS-CoV-2-infected NHEB and A549 cell lines, as well as the uninfected group, for a total of 110 samples. The SARS-004 group indicated that SARS-CoV-2 infected NHBE cells at a multiplicity of infection (MOI) of 2, CoV-002 group indicated that SARS-CoV-2 infected A549 cells at an MOI of 0.2, and the control group were the corresponding uninfected cell lines. For the deregulation gene expression assay, the ‘limma’ package of R software was employed to test the DEGs. mRNAs with log2-fold change (|log2-FC|>1.2, *p* < 0.01) were considered to be differentially expressed mRNAs.

### Identification of core Sub-network from compound-target network

To identify core targets from the compounds-target network, the overlap of DEGs and targets was extracted. Eight overlapping targets were identified. We selected eight targets and their neighbours (only compounds) as the core sub-networks.

### Construction of gene enrichment analysis

To better understand the processes associated with COVID-19 and TCM treatment, we performed Gene Ontology (GO) and Kyoto Encyclopaedia of Genes and Genomes (KEGG) pathway enrichment analyses. GO enrichment analysis provides three structured networks of defined terms to describe gene attributes. Enriched GO terms were classified according to biological process, molecular function, and cellular component. KEGG (http://www.genome.jp/kegg/) is a database for the large-scale systematic analysis of molecular interaction networks of genes or proteins. DAVID, an online bioinformatics resource, consists of an integrated biological knowledge-based analysis tool that systematically extracts biological data from large gene or protein lists. DAVID was applied to determine over-represented GO terms and KEGG pathways with thresholds of count >5 and *p* < 0.05; it also analysed COVID-19-related pathways and GO terms. A histogram of the top ten items was generated by the online mapping tool Bioinformatics (http://www.bioinformatics.com.cn). Venn diagrams and bubble graphs of the top fifteen items were generated using the free online analysis tool OmicShare (http://www.omicshare.com/tools). Significant KEGG pathway terms were mapped to a bubble graph. The larger and higher bubbles represent highly significantly enriched pathway terms.

### Protein-protein interaction network analysis

Protein-protein interaction (PPI) networks include information on the biological processes and molecular functions of cells. We used STRING Version 9.1 (http://www.string-db.org) and Cytoscape software 3.7.2 (http://cytoscape.org/) for recurring instances of neighbouring genes to predict interactions and visualise networks, respectively. To identify crucial relationships in the PPI network, potentially overlapping modules that were densely connected were subsequently identified using the Molecular Complex Detection (MCODE) plugin in Cytoscape. The MCODE plugin was used to reanalyse the clusters among the networks according to k-core = 2. The significance threshold was set at *p* < 0.01.

### Network construction of KEGG pathway

To better analyse the holistic mechanism of Chinese herbs in COVID-19 treatment, the sub-network pathways were compiled using the following procedures: first, all targets of Chinese herbs in COVID-19 treatment were submitted to the KEGG Search Pathway (https://www.genome.jp/kegg/tool/map_pathway1.html). Second, based on the mechanism of COVID-19, multiple pathways were integrated and overlapped according to the cross-talk targets in these maps. Finally, based on the cross-talk of the pathways, we constructed a target-pathway network of TCM treatment.

### Network construction

We established a network analysis of the relevant compound targets. The components of the target networks for the herbs were constructed using Cytoscape. The nodes in each network were evaluated based on three indices: degree, node betweenness, and node closeness. The degree indicates the number of edges between a single node and the other nodes in a network. Node closeness represents the inverse of the sum of the distances from one node to the other nodes. The importance of a node in a network is indicated by the values of these indices, with higher values indicating greater importance.

### Component-molecular target docking

We downloaded the three-dimensional structure of SARS-CoV-2 related proteins from the RSCB PDB database (https://www.rcsb.org/), then removed ligands and non-protein molecules (such as water molecules) in the target proteins using Discovery Studio 2020. We downloaded the two-dimensional structures of the compounds from the PubChem database (https://pubchem.ncbi.nlm.nih.gov/). We used Pyrx software to calculate and export the corresponding 3 D structures structure by minimising energy. Finally, Vina was used for docking studies.

### Cells

A549 cells were cultured in DMEM containing 1% penicillin-streptomycin and 10% foetal bovine serum (Gibco, TX, USA) in an incubator at 37 °C and 5% CO_2_, and the medium was changed every two days in one week. After cell adhesion, logarithmic growth phase cells were used for subsequent experiments.

DMSO was used as a solvent for folic acid in the experimental groups and was added to the blank group without folic acid as a control. Cells were grouped into a blank group (0.1% DMSO), low-concentration folic acid treatment group (0.2 μmol/L), medium-concentration folic acid treatment group (0.4 μmol/L), and high-concentration folic acid treatment group (0.8 μmol/L).

### Plasmid construction

The N full-length vector (SARS-CoV-2 N-FL) was provided by Prof. Shou-Deng Chen of the Fifth Hospital of Sun Yat-Sen University, and subcloned into the pCMV-N-Flag vector using BamHI and XhoI (Kang et al. 2020). The coronavirus N protein acts as a viral suppressor of RNA silencing in mammalian cells (Cui et al. 2015). The shRNA was designed based on the sequence of *mCherry*, named sh-mCherry, and cloned into pLKO1. The main detection index in this experiment was *mCherry* contained by pCDH-mCherry. The shRNA oligonucleotide primers are listed in Table 2.

**Table 2.** Sequences of primers used in RNAi and qRT-PCR.

Name	Sequence
q-mCherry-F	AAGCTGAAGGTGACCAAGG
q-mCherry-R	TTGGAGCCGTACATGAACTG
sh-mCherry-F	CCGGGGACTACACCATCGTGGAACATTCAAGAGATGTTCCACGAT GGTGTAGTCCTTTTTTGGTAAC
sh-mCherry-R	AATTGGTACCAAAAAAGGACTACACCATCGTGGAACATCTCTTGAA TGTTCCACGATGGTGTAGTCC
q-GAPDH-F	AATCCCATCACCATCTTCCAG
q-GAPDH-R	AAATGAGCCCCAGCCTTC

### Transfections

The plasmid was transfected into A549 cells with X-tremeGENE HP DNA transfection reagent (Roche, Basel, Switzerland) using 0.8 μg of each plasmid. The culture medium was replaced with fresh culture medium 6-12 h post transfection, and plasmid expression assays were performed 72 h after transfection.

The transfection groups were as follows: (A) pCDH-mCherry; (B) pCDH-mCherry and sh-mCherry; (C) pCDH-mCherry, sh-mCherry, and pCMV-N-Flag. Each group was divided into a control group and three experimental groups, treated with 0.1% DMSO, and 0.2, 0.4, 0.8 μmol/L folic acid separately.

### Total RNA extraction

Total RNA was extracted using the E.Z.N.A. Total RNA Kit I (Omega Bio-Tek, GA, USA) according to the manufacturer's instructions. The extracted mRNA was reverse transcribed to DNA (cDNA) using the PrimeScript RT Kit (TaKaRa, Dalian, China).

### Reverse transcription PCR (RT-PCR) and quantitative real-time PCR (qPCR)

RT-PCR was used to detect the relative *mCherry* mRNA expression, and *GAPDH* was used as an internal reference. Detection was performed with a SYBR Premix Ex Taq II Kit (TaKaRa) and analysed by the CFX96 Real-Time System (Bio-Rad Laboratories, CA, USA). The cycling program was set to 94 °C for 10 s, 40 cycles of 95 °C (5 s), and 60 °C (40 s). Each assay was repeated thrice. The quantitative primers for *mCherry* and *GAPDH* are listed in Table 2.

### Western blotting analysis

A549 cells were washed twice with PBS and lysed with 100 μL of immunoprecipitation buffer (Beyotime, Shanghai, China) containing 1% phenylmethanesulfonyl fluoride protease inhibitor (Beyotime). The proteins were extracted and resolved using 12% sodium dodecyl sulfate-polyacrylamide gel electrophoresis. Rabbit anti-FLAG (Abcam, Cambridge, UK), mouse anti-mCherry (Abcam) and mouse anti-β-actin (Beyotime) were used for immunoblotting analysis. β-actin-1 was used as the reference. Horseradish peroxidase-conjugated goat anti-rabbit and goat anti-mouse immunoglobulin G (Beyotime) were used as secondary antibodies.

### Immunofluorescence assay

The cells were washed three-times with PBS and incubated with sealing fluid at 37 °C for 90 min. The cells were then incubated with rabbit anti-FLAG (Abcam) and mouse anti-mCherry (Abcam) for 1 h at 37 °C. After incubation, cells were washed with PBS six times, incubated with Alexa Fluor 555-conjugated goat anti-mouse IgG and Alexa Fluor 488-conjugated goat anti-rabbit IgG for 1 h, counterstained with 0.1 μg/mL Hoechst (Beyotime), and washed again with PBS six times. Finally, cells were observed under a confocal microscope. Cell fluorescence signals and numbers were detected by flow cytometry, and the data were analysed using CellQuest Pro.

### Statistical analysis

All data are expressed as mean ± S.D. Differences between groups were determined by paired Student’s *t*-test to determine whether they were statistically significant. Statistical significance was defined as *p* < 0.05, *n* = 3.

## Results

### Identification of effective compounds and target genes of COVID-19 with clinical TCM

A total of 374 active components and 8355 target genes were obtained by analysing and summarising the active drug components. *Amygdalus communis* Vas (Rosaceae), *Bupleuri chinense* DC (Apiaceae), *Scutellaria baicalensis* Georgi (Lamiaceae), *Citrus reticulata* L. (Rutaceae), *Pogostemon cablin* (Blanco) Benth (Lamiaceae), *Ephedra sinica* Stapf (Ephedraceae), *Notopterygium incisum* Ting ex H. T. Chang (Apiaceae), *Atractylodes lancea* (Thunb.) DC (Asteraceae), *Magnolia officinalis* Rehd et Wils. (Magnoliaceae), *Areca catechu* L. (Arecaceae), *Amomum tsaoko* Crevost et Lemarie (Zingiberaceae), *Anemarrhena asphodeloides* Bunge (Asparagaceae), *Forsythia suspensa* (Thunb.) Vahl (Oleaceae), *Glycyrrhiza uralensis* Fisch (Fabaceae), and *Arum ternatum* Thunb. (Araceae) were repeated in 10 different prescriptions. Furthermore, the correlation analysis of these effective compounds revealed that a hub effective component MOL000422 (kaempferol, C_15_H_10_O_6_, SAS = 76.85), was present in the 10 prescriptions (Figure 1(A) and Table 3). There were 77 effective component types associated with prescriptions 1, 3, 4, 6, 7, 9, and 10. Additionally, TCM target genes were collected for GO and KEGG pathway analysis. The results imply that target genes are significantly involved in positive regulation of transcription from RNA polymerase II promoter, nucleus, and protein binding categories (Figure 1(B)), and are mainly associated with pathways involved in cancer, tumour necrosis factor (TNF) signalling, and osteoclast differentiation (Figure 1(C)). Effective compounds and target genes were identified through integrated bioinformatics analysis based on TCMSP, TTD, PubChem, CTD, and GEO datasets (Supplemental Figure 1).

Figure 1.Effective ingredient and target gene analysis of TCM. (A) Venn analysis of effective compounds in TCM. The horizontal bar chart shows the number of active compounds contained in each pharmacy. The longitudinal bar chart shows the number of identical compounds contained in several pharmacies. (B) Gene Ontology (GO) analysis of target genes. (C) KEGG pathways of target genes.

**Figure:**
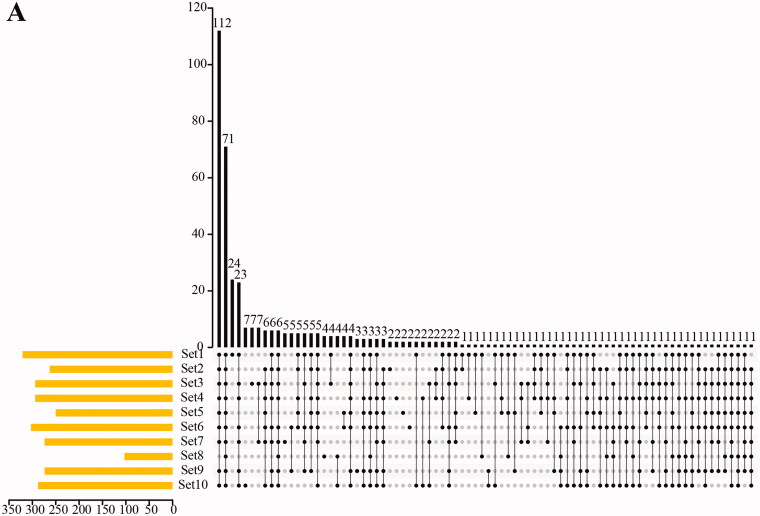

**Figure:**
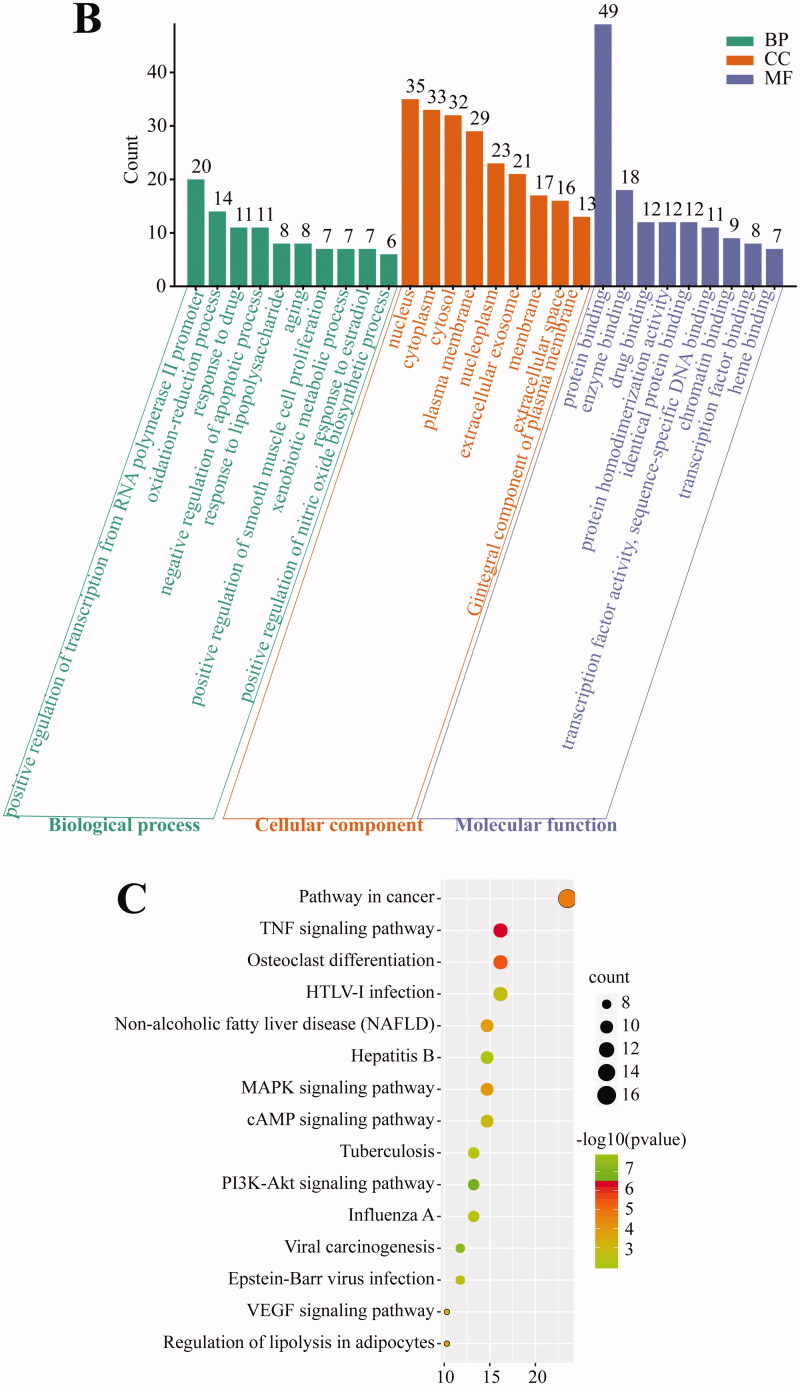

**Table 3.** Statistical results of Venn analysis of effective compounds.

Names	total	elements
user_list1 user_list10 user_list2 user_list3 user_list4 user_list5 user_list6 user_list7 user_list8 user_list9	1	MOL000422
user_list1 user_list10 user_list2 user_list3 user_list4 user_list5 user_list6 user_list7 user_list9	4	MOL000006 MOL000359 MOL004328 MOL000098
user_list1 user_list10 user_list2 user_list3 user_list4 user_list6 user_list7 user_list9	7	MOL004903 MOL004841 MOL000211 MOL002311 MOL000354 MOL005017 MOL004908
user_list1 user_list10 user_list2 user_list3 user_list4 user_list5 user_list6	1	MOL000449
user_list1 user_list10 user_list3 user_list4 user_list6 user_list7 user_list9	77	MOL005016 MOL004990 MOL005001 MOL004978 MOL004959 MOL004879 MOL000417 MOL004884 MOL004857 MOL004904 MOL004911 MOL004828 MOL005020 MOL000239 MOL004835 MOL004863 MOL004949 MOL004945 MOL004805 MOL005000 MOL004985 MOL001484 MOL004912 MOL004838 MOL004957 MOL004808 MOL001792 MOL000392 MOL004833 MOL004898 MOL004815 MOL004829 MOL004864 MOL004913 MOL005012 MOL004885 MOL004961 MOL004891 MOL005003 MOL000500 MOL005018 MOL004993 MOL004811 MOL004996 MOL003656 MOL004827 MOL004820 MOL004856 MOL004882 MOL004824 MOL004980 MOL004848 MOL005008 MOL004814 MOL004935 MOL004866 MOL004907 MOL002565 MOL000497 MOL004974 MOL004914 MOL004883 MOL004855 MOL004806 MOL004988 MOL004991 MOL004810 MOL004941 MOL004849 MOL003896 MOL004910 MOL005007 MOL004948 MOL004924 MOL004915 MOL004989 MOL004966
user_list1 user_list2 user_list3 user_list5 user_list6 user_list7	1	MOL000173
user_list1 user_list2 user_list4 user_list5 user_list6 user_list9	8	MOL005918 MOL005923 MOL005573 MOL005916 MOL005911 MOL002879 MOL005922 MOL005921
user_list1 user_list2 user_list4 user_list5 user_list6	16	MOL001755 MOL000492 MOL005190 MOL001771 MOL004798 MOL005043 MOL001494 MOL011319 MOL007214 MOL002823 MOL010489 MOL005842 MOL004576 MOL002881 MOL000358 MOL010788
user_list1 user_list10 user_list3 user_list6 user_list9	1	MOL002776
user_list1 user_list2 user_list4 user_list6	7	MOL004355 MOL002211 MOL000953 MOL012922 MOL007207 MOL005030 MOL010921
user_list1 user_list3 user_list6 user_list9	1	MOL002714
user_list1 user_list4 user_list5 user_list9	1	MOL005828
user_list2 user_list3 user_list5 user_list6	1	MOL005980
user_list1 user_list2 user_list9	1	MOL000072
user_list1 user_list3 user_list4	2	MOL004609 MOL002933
user_list1 user_list3 user_list7	1	MOL001458
user_list1 user_list5 user_list9	2	MOL005815 MOL005100
user_list1 user_list6 user_list9	1	MOL000519
user_list2 user_list3 user_list5	1	MOL002032
user_list3 user_list4 user_list6	1	MOL002235
user_list3 user_list6 user_list7	1	MOL001924
user_list10 user_list6 user_list9	1	MOL000433
user_list1 user_list3	23	MOL000525 MOL012245 MOL002322 MOL002917 MOL012266 MOL013187 MOL000228 MOL002915 MOL012246 MOL002934 MOL002927 MOL001689 MOL000552 MOL002937 MOL002914 MOL002932 MOL008206 MOL002925 MOL002928 MOL002909 MOL004598 MOL002910 MOL002913
user_list1 user_list4	4	MOL013277 MOL013279 MOL001803 MOL001798
user_list3 user_list4	6	MOL004083 MOL001781 MOL007274 MOL004112 MOL005229 MOL007401
user_list3 user_list7	5	MOL004497 MOL004528 MOL003370 MOL000791 MOL004373
user_list4 user_list6	3	MOL002280 MOL002259 MOL002268
user_list6 user_list9	4	MOL000379 MOL000380 MOL000371 MOL000387
user_list10 user_list7	1	MOL002222
user_list8 user_list9	1	MOL005321
user_list10 user_list9	1	MOL007059
user_list1	32	MOL013428 MOL000856 MOL002818 MOL000322 MOL012140 MOL002786 MOL001559 MOL002962 MOL002563 MOL010013 MOL010028 MOL001736 MOL012141 MOL010017 MOL005465 MOL010014 MOL000849 MOL000351 MOL009849 MOL010006 MOL000831 MOL000103 MOL010015 MOL010004 MOL010007 MOL000822 MOL010003 MOL001735 MOL000073 MOL000862 MOL001558 MOL001460
user_list2	3	MOL005619 MOL001040 MOL002605
user_list3	3	MOL002309 MOL006992 MOL001810
user_list4	3	MOL013288 MOL005849 MOL013281
user_list6	2	MOL000471 MOL002288
user_list7	2	MOL002668 MOL007662
user_list8	8	MOL003648 MOL005344 MOL000787 MOL005356 MOL005384 MOL005530 MOL008457 MOL003137
user_list9	3	MOL008400 MOL008411 MOL002140
user_list10	43	MOL007069 MOL007156 MOL007101 MOL002651 MOL007150 MOL003857 MOL007124 MOL007155 MOL008957 MOL007068 MOL007108 MOL007036 MOL007119 MOL007058 MOL007088 MOL003759 MOL007048 MOL007151 MOL007079 MOL008974 MOL003847 MOL007130 MOL007152 MOL007049 MOL007061 MOL007125 MOL007093 MOL008978 MOL007085 MOL006630 MOL007122 MOL007879 MOL007127 MOL001601 MOL007070 MOL007094 MOL007045 MOL007071 MOL007154 MOL007111 MOL007098 MOL007041 MOL008992

### Screening of joint effective compounds and drug target genes (DTGs) by combined analysis of TCM and CWM

**Figure 2.**
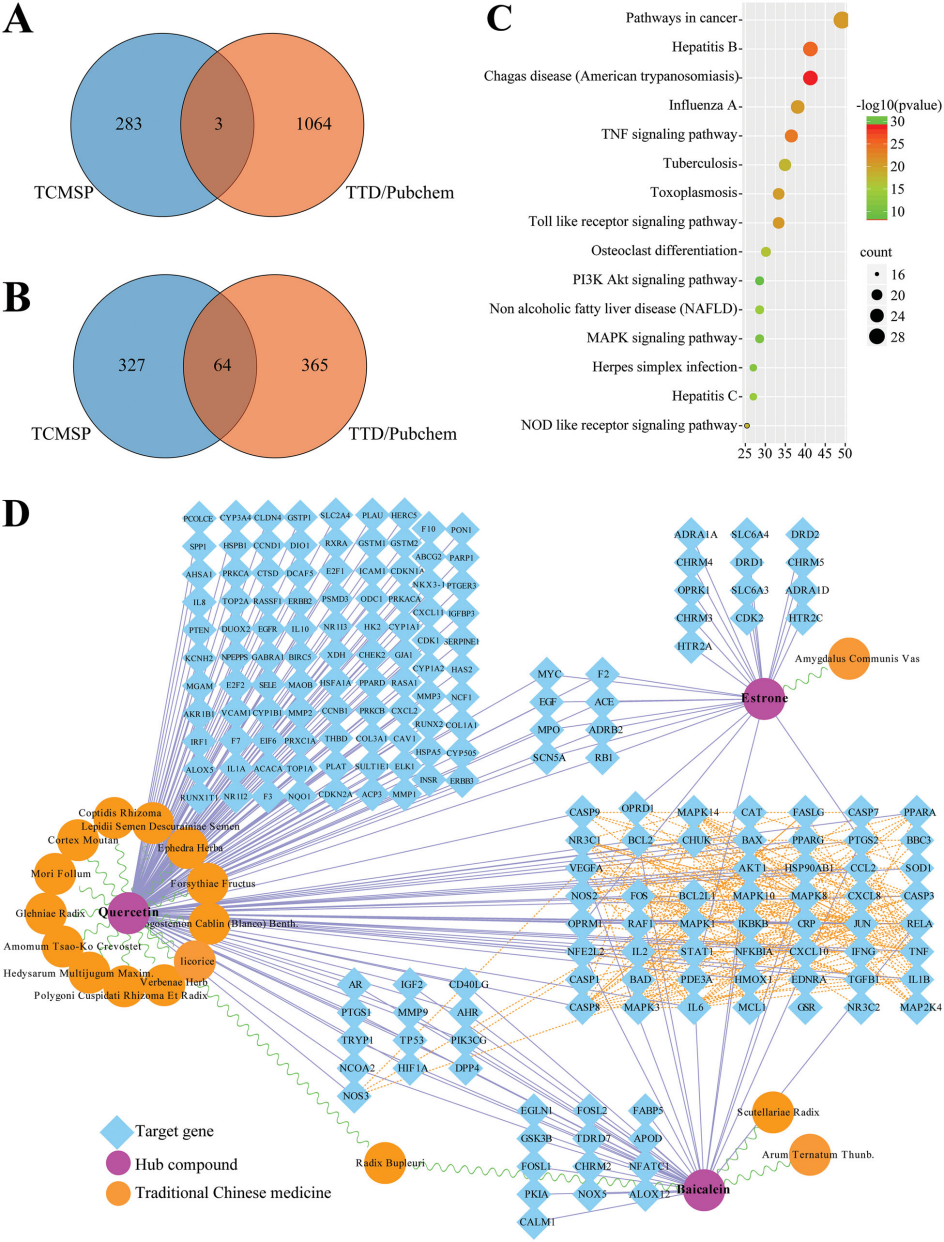
Integrated analysis of COVID-19 clinical TCM and CWM. (A) Venn analysis of effective compounds based on TCMSP, TTD, and PubChem databases. (B) Venn analysis of drug target genes based on TCMSP, TTD, and PubChem. (C) KEGG pathways of target genes. (D) Network of TCM-compounds-target genes.

We first constructed Venn diagrams of TCM and CWM compounds and target genes based on the TCMSP and TTD/PubChem databases, and we found three hub compounds (quercetin, SAS = 75.20; oestrone, SAS = 66.38; baicalein, SAS = 77.75) and 64 target genes (Figure 2(A,B)). Hub target genes were significantly involved in pathways in cancer and hepatitis B, according to KEGG pathway analysis (Figures 2(C)). Moreover, Cytoscape was used to construct the regulatory network of Traditional Chinese Medicine-Hub compound-Target genes (Figure 2(D)), which indicated that quercetin had more complex network systems and oestrone had fewer target genes. *MAPK14*, *MAPK3*, and *MCL1* connected quercetin and baicalein, and *OPRM1*, *ADRB2*, *SCN5A*, *EGF,* and *ACE* connected oestrone and quercetin.

### Identification of DEGs induced by SARS-CoV-2 infection based on GEO database: GSE147507

**Figure 3.**
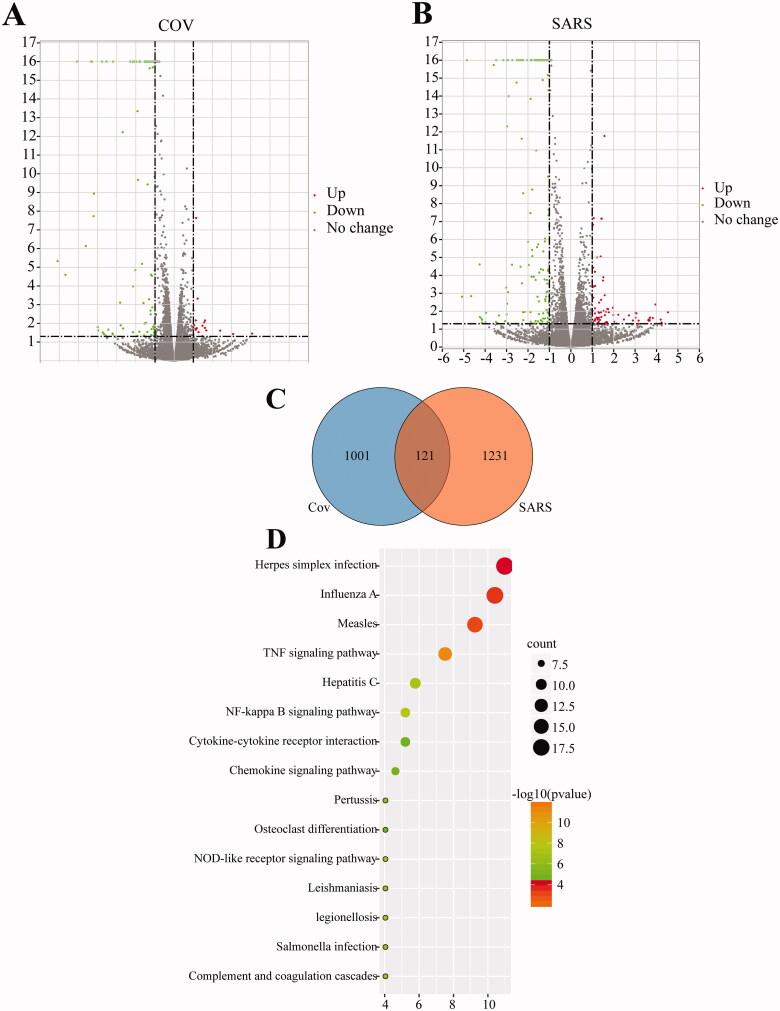
Identification of DEGs in GSE147507 dataset. (A) Volcano plots of the distribution of DEGs in the CoV-set. (B) Volcano plots of the distribution of DEGs in SARS-set. (C) Venn analysis of DEGs in CoV- and SARS-sets. (D) KEGG pathways of intersection genes.

The results showed that there were 1177 DEGs in A549 cells, and 1407 in NHBE cells. The volcano plots of DEGs among CoV-set and SARS-set are shown in Figure3(A,B). Venn diagram analysis reveals that eight upregulated genes and 113 downregulated genes are the same in the two cell types (Figure 3(C) and Table 4).

**Table 4.** Total number of differentially expressed genes in GSE147507 dataset.

Category	Numbers of DEGs		
	Cov002_mock_ vs_Co002_CoV2	SARS004_mock_ vs_SARS004_CoV2	Venn
Up-regulated	349	677	8
Down-regulated	828	730	105
Total number	1177	1407	113

The DEGs KEGG pathway enrichment shows that DEGs are significantly involved in the pathway of herpes simplex infection, influenza A, measles, and TNF signalling pathways (Figure 3(D)). The above results indicate that SARS-CoV-2 infection markedly downregulated the host immune response.

### Identification of key hub DEGs by integrating internet-pharmacology and GEO database

To further screen molecular targets for COVID-19 regulation, and provide targets for exploring the mechanism of interaction between the virus and host, as well as screening new drugs, we conducted molecular docking between DTGs and DEGs induced by SARS-CoV-2 infection, screening core pivotal genes. Venn analysis was carried out on the target genes of 10 TCM prescriptions and DEGs induced by virus infection (Figure 4(A)), and common associated genes were identified. These genes were down regulated by viral infection in both CoV and SARS groups (Figure 4(B,C)).

Figure 4.Molecular docking of DTG and DEGs. (A) Venn analysis of DTG in different TCM prescriptions and DEGs. (B) The fold change value of DTG-DEGs genes in SARS-set. The integrated genes were screened based on TCM target genes and DEGs in SARS-set, and the fold change of hub genes were analysed in GSE147507. (C) The fold change value of DTG-DEGs genes in CoV-set. The integrated genes were screened based on TCM target genes and DEGs in CoV-group, and the fold change of hub genes were analysed in GSE147507. (D) The fold change value of TTD-TCMSP-GEO genes in SARS-group. (E) The fold change value of TTD-TCMSP-GEO genes in CoV-group. *Indicates that the gene is present in both CoV-set and SARS-set. (F) The chemical interaction of key hub genes. We screened chemical interaction of genes present in Venn analysis of TTD-TCMSP-GEO and both SARS-group and CoV-group.

**Figure:**
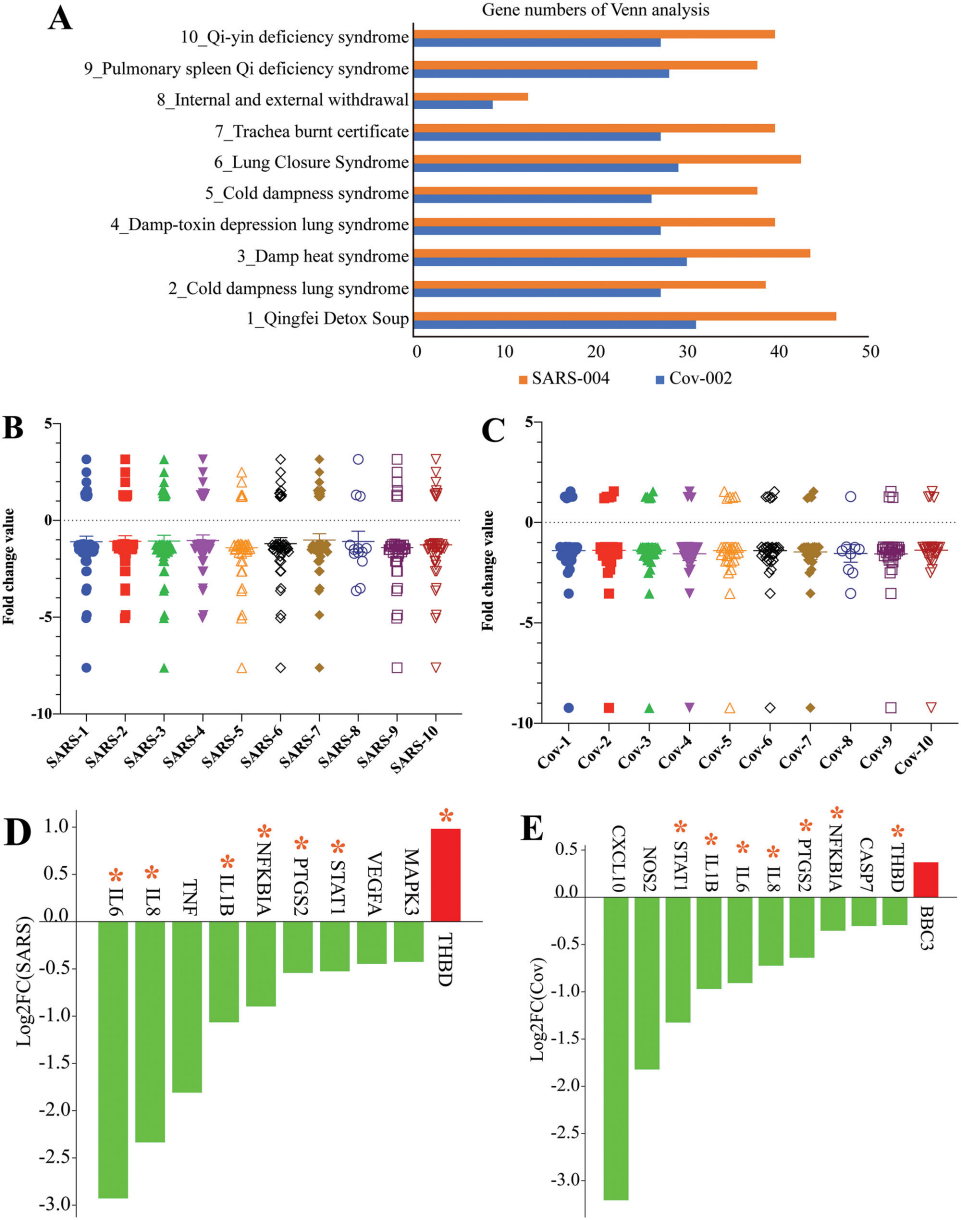

**Figure:**
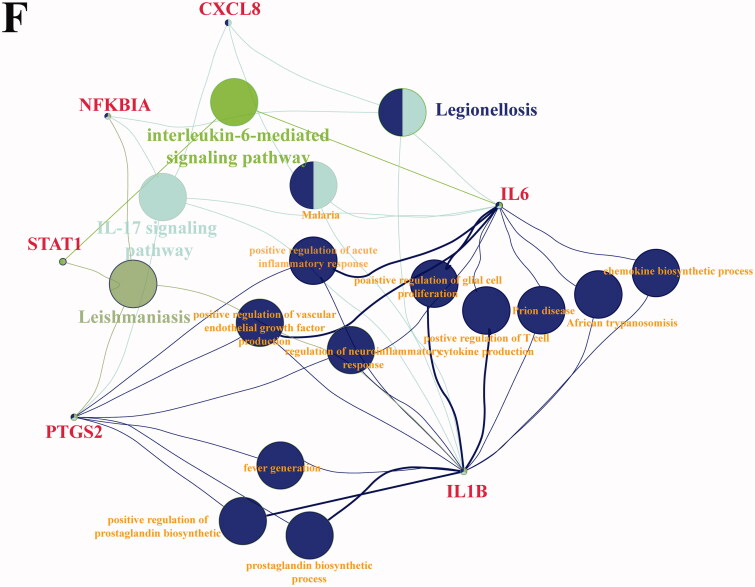

Additionally, based on the TTD, GEO, and TCMSP databases, DTGs and the DEGs induced by the virus were analysed, and 14 core pivotal genes were obtained. *IL6*, *IL8*, *IL1B*, *NFKBIA*, *PTGS2*, *STAT1*, and *THBD* were all present in the CoV and SARS groups. With the exception of *THBD*, these genes were downregulated by viral infection in both groups (Figure 4(D,E)). KEGG pathway enrichment of these genes is shown in Figure 4(F). Finally, the interaction compounds of core hub genes were analysed using the CTD, and two common compounds, folic acid (SAS = 99.00) and ozone, were screened (Supplemental Figure 2). Folic acid and ozone can downregulate and upregulate core hub gene expression, respectively.

### Identification of component-molecular target docking

The docking score was normalised for molecular weight (DSnorm, KJ/mol); values < −5.0 are considered the screening condition (Sen-nan et al. 2018). Docking results are shown in Table 5. The lower the docking score is, the stronger the binding between compound and molecular target. This means that the compound has a greater ability to make a difference. Among the six compounds (kaempferol, quercetin, baicalin, oestrone, folic acid, and ozone), quercetin, baicalin, oestrone, and folic acid were able to bind spontaneously to the hub gene. The average docking score of folic acid was the lowest. Folic acid also has the strongest binding ability to hub genes, which means it has the most potential as a targeted therapy.

**Table 5.** Docking score of effective compounds to hub genes.

Compounds	Chemical formula	IL1B	IL6	IL8	NFKBIA	PTGS2	STAT1	Average
Baicalein	C_15_H_10_O_5_	−7.1	−6.9	−6.1	−7.9	−8.8	−6.2	−7.2
Oestrone	C_18_H_22_O_2_	−6.4	−7.4	−6.5	−8.1	−8.4	−6.5	−7.2
Quercetin	C_15_H_10_O_7_	−7.1	−7.0	−6.1	−8.4	−9.1	−6.5	−7.4
Folic acid	C_19_H_19_N_7_O_6_	−7.2	−6.8	−6.5	−8.5	−10.0	−6.8	−7.6

**Figure 5.**
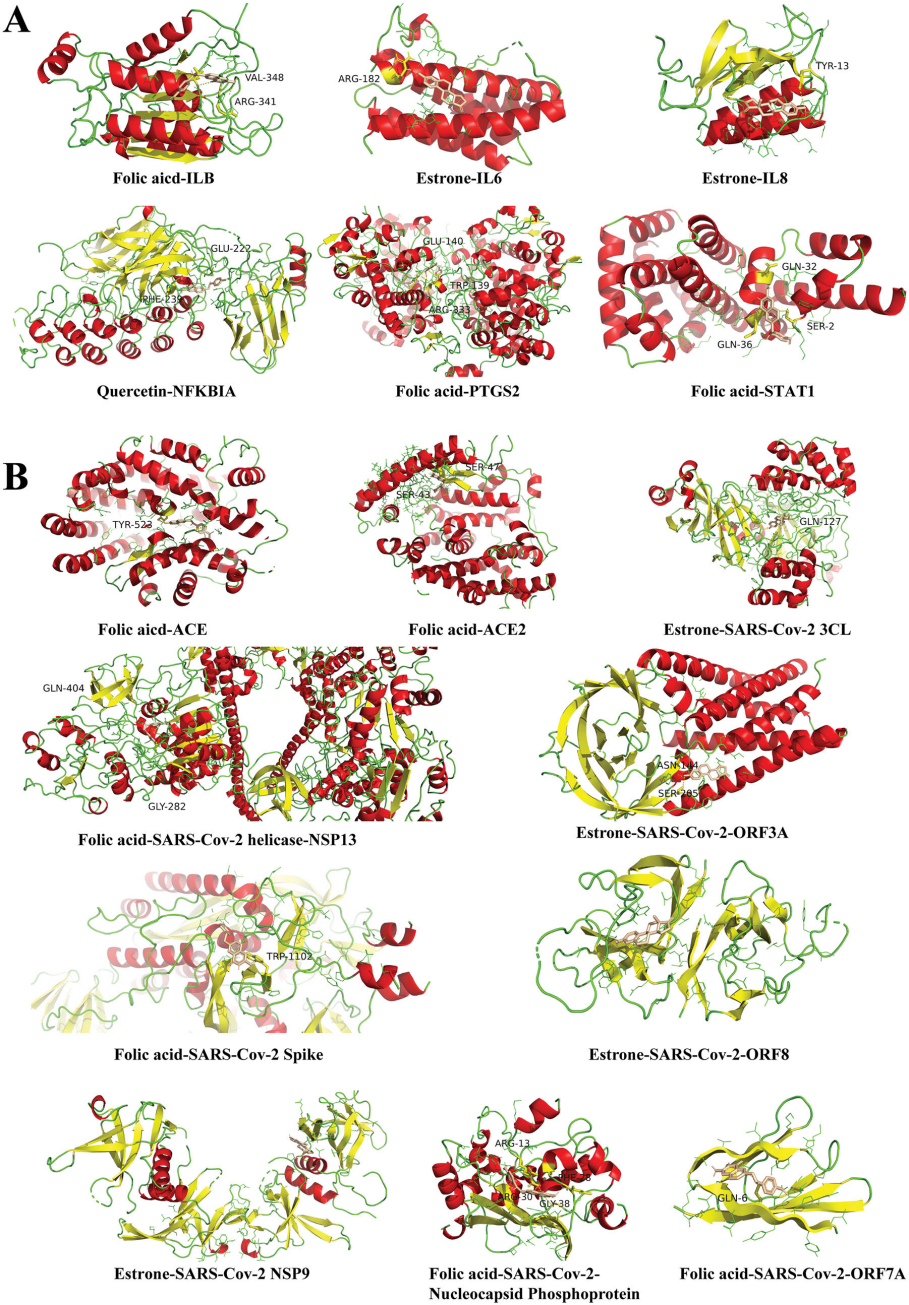
Molecular docking of effective compounds to hub genes and SARS-CoV-2-related genes. (A) The 3 D pattern that shows the hub genes of effective compounds on the associated proteins. (B) The 3 D pattern that shows the molecular docking of the SARS-CoV-2-related genes and effective compounds.

Next, the compounds were docked with SARS-CoV-2-related genes, and the results of docking score < −5 are shown in Table 6. Folic acid is the compound that binds the gene most strongly. Among SARS-CoV-2-related genes, SARS-COV-2 3CL, SARS-CoV-2 Nucleocapsid Phosphoprotein, SARS-COV-2 ORF3a, and SARS-CoV-2 ORF8 all show good binding properties to compounds. Among them, SARS-CoV-2 Nucleocapsid Phosphoprotein is the most tuberculous. Based on the above results, we hypothesised that folic acid can act on SARS-CoV-2 N. For each gene, the combination with the lowest docking score was selected for visual processing (Figure 5).

**Table 6.** Docking score of effective compounds to SARS-Cov-2 related genes.

Compounds	Baicalein	Oestrone	Quercetin	Folic acid
Chemical formula	C_15_H_10_O_5_	C_18_H_22_O_2_	C_15_H_10_O_7_	C_19_H_19_N_7_O_6_
ACE	−7.9	−8.3	−8.0	−8.6
ACE2	−6.5	−7.0	−6.0	−7.5
SARS-Cov-2 3CL	−7.8	−8.3	−8.0	−7.7
SARS-Cov-2 helicase-NSP13	−7.6	−7.4	−7.0	−7.9
SARS-Cov-2 Spike	−5.8	−5.5	−5.6	−6.6
SARS-Cov-2 NSP9	−7.3	−7.4	−7.0	−7.4
SARS-Cov-2 Nucleocapsid Phosphoprotein	−8.0	−7.8	−7.8	−8.3
SARS-Cov-2 ORF3a	−7.7	−8.0	−7.5	−7.6
SARS-Cov-2 ORF7a	−5.1	−5.8	−5.6	−6.5
SARS-Cov-2 ORF8	−7.6	−7.9	−7.6	−7.6
Average	−7.1	−7.3	−7.0	−7.6

### Folic acid antagonises the inhibitory effect of SARS-CoV-2 N protein on the RNA interference pathway

The *mCherry* expression level in each treatment group was compared with the mRNA level by RT-qPCR (Figure 6(A)). The results of the DMSO-treated groups showed that RNA interference (RNAi) decreased *mCherry* expression by 81.7% (*p* < 0.001). In contrast, *mCherry* expression was decreased by 8.0% in the presence of SARS-CoV-2 N protein, indicating that the SARS-CoV-2 N protein inhibited the RNAi process. With the increase of folic acid treatment concentration to 0.2, 0.4, and 0.8 μmol/L, *mCherry* expression decreased 19.2%, 50.0%, 80.1%, respectively, demonstrating that *mCherry* expression could be successfully inhibited by addition of folic acid and SARS-CoV-2 N, indicating that RNAi was restored. Western blotting results showed that in the DMSO control group, the *mCherry* level decreased significantly after interference, and there was no significant difference in the group in which SARS-CoV-2 N and shRNA fragments were simultaneously added, indicating that SARS-CoV-2 N inhibited the RNAi pathway in cells. After cell treatment with folic acid, the mCherry protein level in the SARS-CoV-2 N group decreased, and the degree of decrease was proportional to the folic acid concentration, indicating the antagonistic effect of folic acid on the function of SARS-CoV-2 N (Figure 6(B)). To visually identify the antagonistic effect of folic acid on SARS-CoV-2 N, we used a fluorescence microscope. While *mCherry* and SARS-CoV-2 N exhibit red and green fluorescence, respectively, blue fluorescence represents the number of cells. We found that there was no significant difference between the group in which SARS-CoV-2 N and shRNA fragments were simultaneously added and the control group after DMSO treatment.

After folic acid treatment, the group in which SARS-CoV-2 N and shRNA were simultaneously added exhibited less red fluorescence than that of the control group (Figure 6(C)). The red cell percentage was counted using flow cytometry, which objectively demonstrated mCherry protein level expression (Figure 6(D)). The results for the DMSO-treated group were the same as those described previously. The mCherry protein expression in the group expressing SARS-CoV-2 N was higher under lower concentrations of folic acid, and the data were significantly different between groups (*p* < 0.05). The results indicate that high concentrations of folic acid antagonise the function of SARS-CoV-2 N and restore the cellular RNAi pathway.

Figure 6.Data statistics of genes expression in the cellular experiment. (A) RT-qPCR results of *mCherry* mRNA level expression in each treated group. (B) Western blotting results of protein-level expression of mCherry and N protein in each treated group. (C) Protein level expression of genes in each treated group, as revealed by immunofluorescence technique. (D) Flow cytometry statistics of protein-level expression of genes in each treated group.

**Figure:**
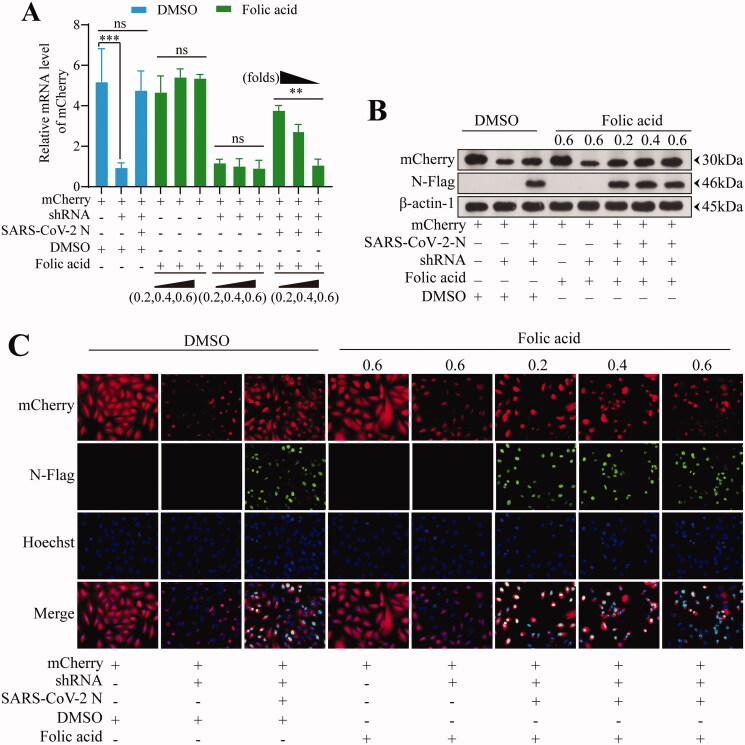

**Figure:**
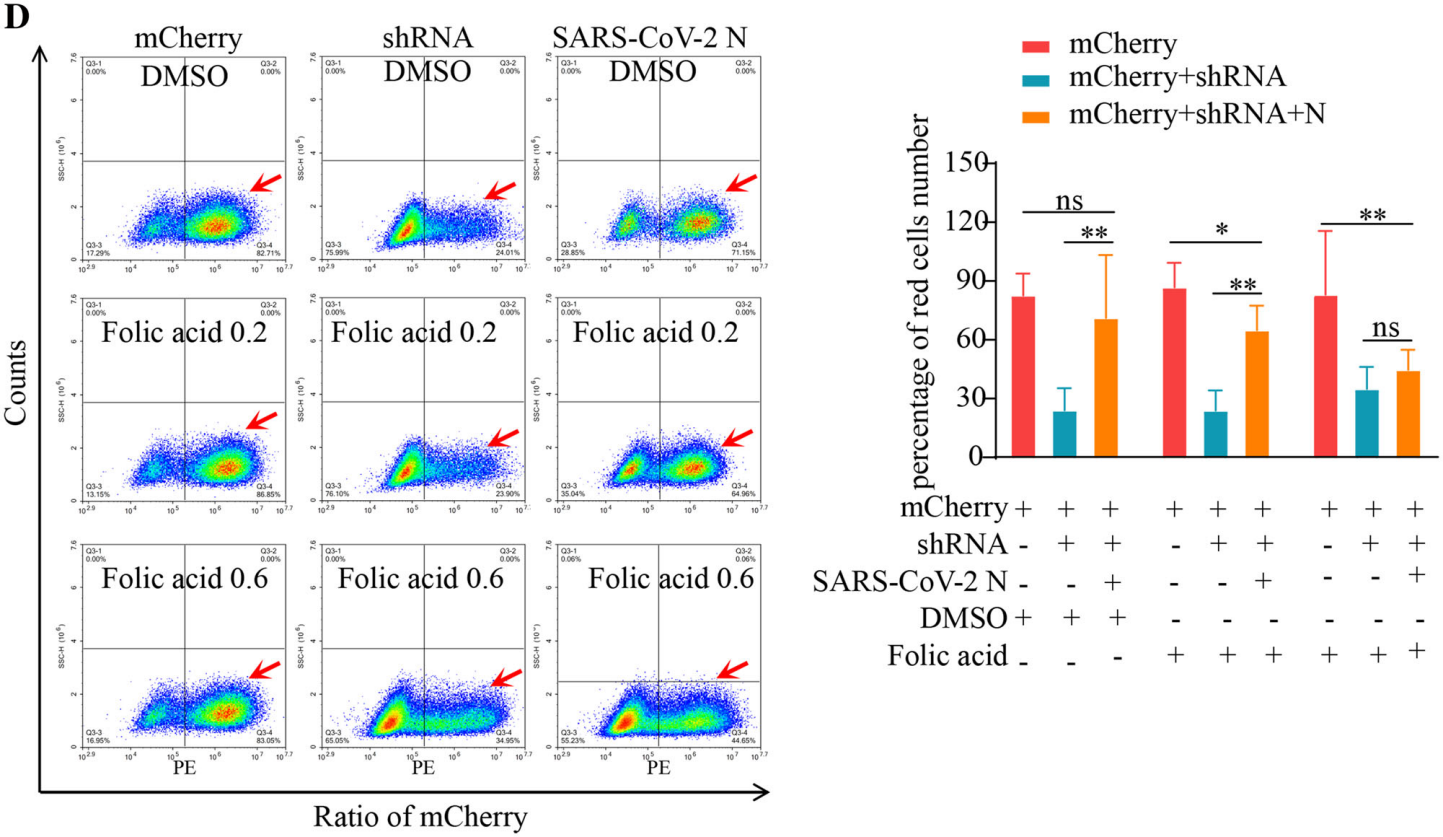

## Discussion

The SARS-CoV-2 infection molecular therapeutic network model was built based on intelligent computer and big data integration bioinformatics. The project primarily relies on database analysis to analyse the interactions and related phenotypes among SARS-CoV-2 drugs and to analyse the key drug targets by correlation analysis. Infected and normal cells were investigated using the GEO database. This research mainly relied on published data, available in the database, to integrate bioinformatics analysis, obtain the key drug targets and potential targets of gene therapy, and explore the mechanisms of effective compounds in COVID-19 treatment.

After analysing the active compounds of the 10 TCM prescriptions, a common core compound, kaempferol, was found. It has been shown that kaempferol has some anti-inflammatory, anti-cough and expectorant effects (Xiaoqing 2017). This indicates that TCM may play a therapeutic role in COVID-19. In addition, GO function analysis was performed on the target TCM sites, and the results showed that the target genes were enriched in the positive regulation of transcription from RNA polymerase II promoter, oxidation-reduction process, and protein binding. KEGG pathway analysis revealed that target genes were mainly involved in cancer pathways, the TNF signalling pathway, and osteoclast differentiation. These terms play a key role in COVID-19 treatment using TCM.

In the association analysis of TCM and CWM, three central compounds were identified; baicalein, oestrone, and quercetin. Baicalein is a flavonoid with antihyperglycemic activity (Dinda et al. 2017). Quercetin is also a flavonoid; it exhibits beneficial effects on inflammation and immunity (Li et al. 2016). Studies have shown that quercetin may decrease the frequency and duration of respiratory tract infections as an effective intervention; however, further research is needed (Aucoin et al. 2020). Natural oestrogens mainly comprise oestradiol, oestrone, and oestriol. It has been shown that oestrogen treatment silences inflammatory reactions and decreases virus titres, leading to improved survival rates in animal experiments (Zsuzsanna 2020). GO analysis was performed on the overlapping fraction of targets obtained from target association analysis of TCM and CWM to obtain target enrichment in the cytosol and protein binding category. KEGG analysis was performed to obtain target enrichment in the pathways of cancer and hepatitis B. The commonality between TCM and CWM for the treatment of COVID-19 has been shown.

In the analysis of DEGs induced by viral infection, upregulated genes, such as *GNPDA1*, were found to be highly expressed in the kidney, as well as *RPAGD*, *ANKH* and *PRODH* in the thyroid, pancreas, and small intestine, respectively, and downregulated genes were highly expressed in the bone marrow, lymph nodes, spleen, lung, caecum, gallbladder, and bladder. Moreover, key genes were expressed at significantly lower levels in pancreatic and liver tissues. It is hypothesised that these genes are key targets related to clinical COVID-19 symptoms such fever, cough, chest tightness, diarrhoea, and sore throat. GO functional analysis of the DEGs revealed that these genes were mostly enriched in the type I interferon signalling pathway, defense response to virus, cytoplasm, cytosol, and protein binding. KEGG analysis revealed that the DEGs were predominantly enriched in herpes simplex infection, influenza A, and TNF signalling pathways. SARS-CoV-2 infection affects the host immune response. It has been shown that, after infection with SARS-CoV-2, uncontrolled inflammatory mediators in the body can cause a cytokine storm, leading to an excessive immune response, consistent with the results of DEGs enrichment analysis (Quirch et al. 2020). In addition, the GO classification results showed that both TCM drug target genes and virus-induced genes were significantly involved in protein binding among molecular functions.

After analysing the drug targets and DEGs separately, we obtained six core hub genes (*IL6*, *CXCL8*, *IL1B*, *NFKBIA*, *PTGS2*, and *STAT1*) by correlating them to establish a disease-target-compound-drug regulatory network. SARS-CoV-2 activates different immune cells that help to secrete the proinflammatory cytokine *IL6* and other inflammatory cytokines, causing a cytokine storm (Chiranjib et al. 2020). Both *IL8* and *IL1B* encode proteins that are cytokines and, like *IL6*, are significantly more abundant in critically ill patients (*p* < 0.05; Chua et al. 2020; Quirch et al. 2020).

Potential molecular target identification is essential for drug repurposing (Skariyachan et al. 2020; Wang 2020; Lokhande et al. 2021). Through the construction of a molecular docking model between hub host genes and hub compounds, it was found that folic acid and proteins ILB, PTGS2, and STAT1 were stable binding, and oestrogen stably bound to IL6 and IL8. Oestrone has good binding properties to proteins, and exogenous oestrogen is recommended as a drug for COVID-19 prevention and treatment (Zsuzsanna 2020). Folic acid may be a potential target drug, as it has a relatively lower binding energy to proteins, suggesting that it might be an effective drug for COVID-19 treatment.

For the precise treatment of COVID-19, we performed molecular docking between drug compounds and viral proteins. The role of SARS-CoV-2 N is indispensable in RNA recognition, replication, transcription of the viral genome and modulation of the host immune response (Shan et al. 2021). The 3CL protease and NSP13 helicase are crucial for viral replication (Keum and Jeong 2012; Fernandes et al. 2019). The S proteins help infusing into the host, and the NSP9 replicase plays a major role in viral replication (Vaishali et al. 2020). The ORF3a protein can activate the NLRP3 inflammasome by promoting TRAF3-dependent ubiquitination of *ASC* (Siu et al. 2019). The ORF7A protein blocks cell cycle progression at the G0/G1 phase via the cyclin D3/pRb pathway (Schaecher et al. 2008). ORF8 can potentially mediate immune suppression and evasion activities (Flower et al. 2021). These proteins play an important role in viral host infection, so they are selected for molecular docking with the effective compounds obtained from the analysis. 3CL protease, ORF3a protein, and ORF8 have low binding energy with the compounds. The binding of SARS-CoV-2 N to folic acid was stable, indicating that it could be targeted for molecular therapy. Together, the above results show that the binding energy between folic acid and SARS-CoV-2 N is the lowest, so it was speculated that SARS-CoV-2 N might be the target of folic acid in COVID-19 treatment.

Folic acid supplementation modulated B lymphocyte responses and improved innate immune proinflammatory and antiviral response molecular pathways (Uribe-Diaz et al. 2022). It has preventive effects on Zika virus-associated poor pregnancy outcomes in immunocompromised mice. Mice treated with folic acid exhibited lower viral burden and better prognostic profiles in the placenta, including reduced inflammatory response and enhanced blood-placenta barrier integrity (Simanjuntak et al. 2020). Folic acid has been shown to display protective effects in pregnant women with COVID-19 (Acosta-Elias and Espinosa-Tanguma 2020), however, the mechanism by which it protects pregnant women during the COVID-19 pandemic has not been determined. The predisposition to HPV infection persistency and cervical dysplasia progression is known to increase due to folic acid deficiency (Abike et al. 2011). Folic acid supplementation suppresses abnormal p16 protein expression and effectively promotes apoptosis and inhibits proliferation in cervical carcinoma cells (Jia et al. 2016).

These studies suggest that folic acid may have broad-spectrum antiviral effects, as well as potential as a novel drug for COVID-19 treatment. However, high folic acid levels in low vitamin B12 states exacerbate neurological problems, according to the expert panel convened by the U.S. National Toxicology Program (NTP) and the Office of Dietary Supplements of the National Institutes of Health (Boyles et al. 2016). Oral folic acid (pteroylglutamic acid) is not generally considered toxic to normal people, but giving it to patients with undiagnosed pernicious anaemia may cause neurological injury (Butterworth and Tamura 1989). At least one study supports the assertion that high serum folate concentrations in older adults with vitamin B12 deficiency may be associated with increased neurodegeneration (Deng et al. 2017). These studies suggest that we should pay close attention to the dose-related side effects of folic acid in subsequent *in vivo* experiments and explore the optimal dosage for clinical treatment.

A cell experiment was designed to assess the role of folic acid in treating COVID-19. A red fluorescent protein derived from mushroom coral, *mCherry* is used to label and trace certain molecular and cellular components. RNAi is an RNA-dependent gene-silencing phenomenon that can be triggered by shRNA, as well as a strong and versatile silencer of various genes (Xin et al. 2012). SARS-CoV-2 N is a structural protein that binds directly to viral RNA and provides stability; it has two RNA-binding domains, and its forms or regulates biomolecular condensates *in vivo* through interactions with RNA and key host cell proteins (Cascarina and Ross 2020). SARS-CoV-2 N encoded by SARS-CoV-2 plays a vital role in inhibiting RNA interference in cells (Carlson et al. 2020; Mu et al. 2020; Yoshimoto 2020). The cell experiment was designed based on these theories. The shRNA expression inhibits expression of the red fluorescent protein *mCherry*, and SARS-CoV-2 N expression inhibits the effect of shRNA-mediated RNAi. The change in *mCherry* expression reflected the effect of folic acid on SARS-CoV-2 N. As a result, after the addition of folic acid, the inhibitory effect of SARS-CoV-2 N on RNAi was weakened, and *mCherry* expression decreased. The result showed that folic acid inhibited the biological activity of SARS-CoV-2 N, suggesting that folic acid might disrupt RNA, and thus have an anti-viral effect. This needs to be further studied in subsequent experiments. It was preliminarily shown that SARS-CoV-2 N could be a target for folic acid in COVID-19 treatment.

## Conclusions

The study identified the pivotal genes and effective compounds through analysis of effective compounds and drug targets of COVID-19 in clinical TCM and CWM. In total, 8355 drug targets were found; these were involved in positive regulation of transcription via RNA polymerase II promoter, nucleus, and protein-binding categories, all associated with the cancer pathway. DEGs from the GEO database and drug targets were correlated, and 113 targets related to SARS-CoV-2 were found. The related targets were involved in the type I interferon signalling pathway, defense response to virus, cytoplasm, cytosol, protein binding, and herpes simplex infection. Folic acid was found to act on SARS-CoV-2 N by molecular docking. The cell experiment showed that folic acid antagonises the inhibitory effect of SARS-CoV-2 N protein on the RNA interference pathway. This study promoted the underlying interaction mechanism between the virus and host, expanded the medicinal scope of folic acid, and provided new insights for new drug development. In our future work, we will study the mechanism of folic acid on SARS-CoV-2 N.

## Supplementary Material

Supplemental Material

Supplemental Material

## Data Availability

The original contributions presented in the study are included in the article/supplementary materials. Further inquiries can be directed to the corresponding authors.
