# Supplementary figures and images for: Folic acid: a potential inhibitor against SARS-CoV-2 nucleocapsid protein
Source: Pharm Biol. 2022 May 20;60(1):862–78. doi: 10.1080/13880209.2022.2063341 (PMC9132477; doi:10.1080/13880209.2022.2063341)

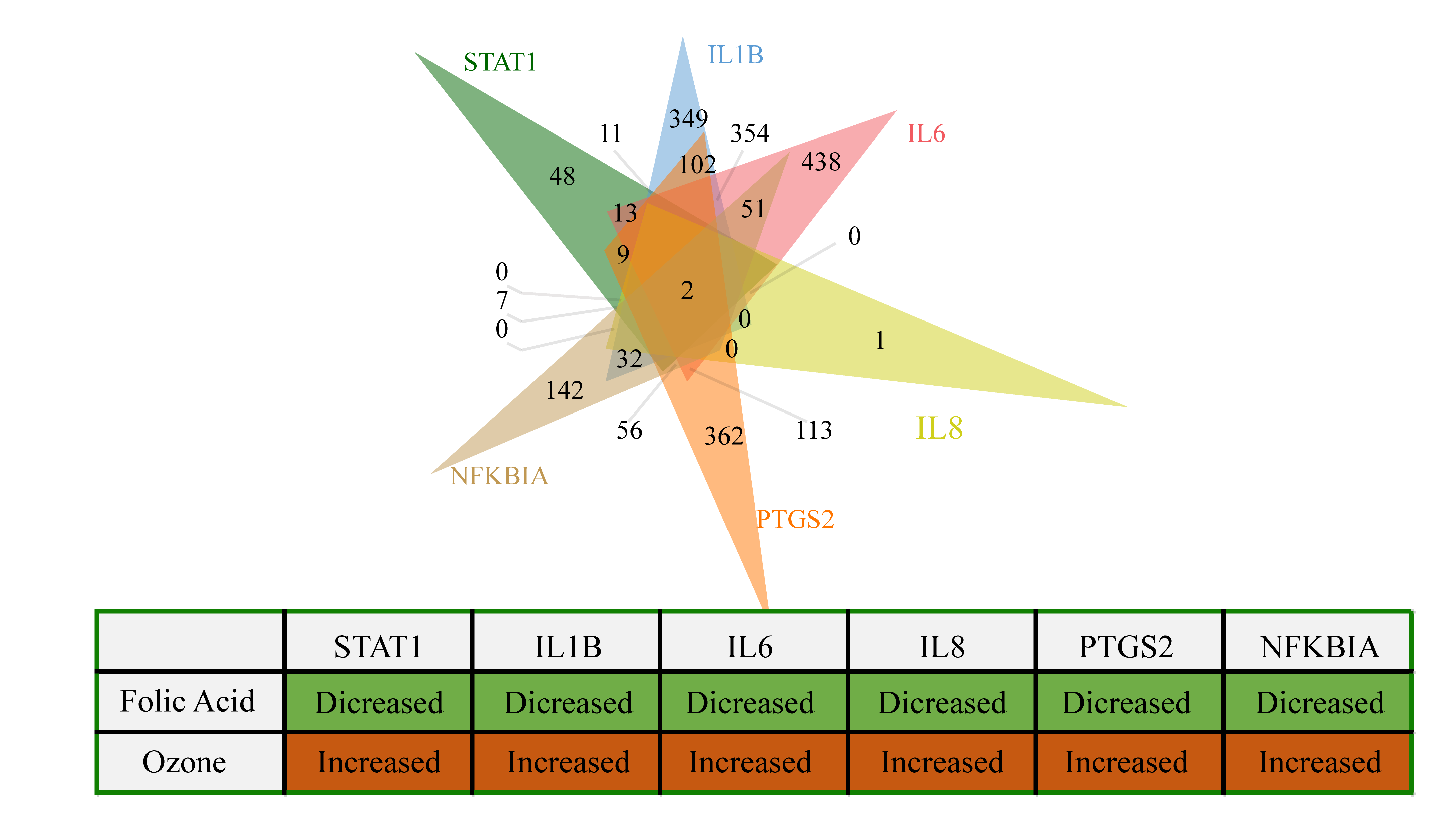

Supplement: Supplemental Material [file IPHB_A_2063341_SM5718.tif]

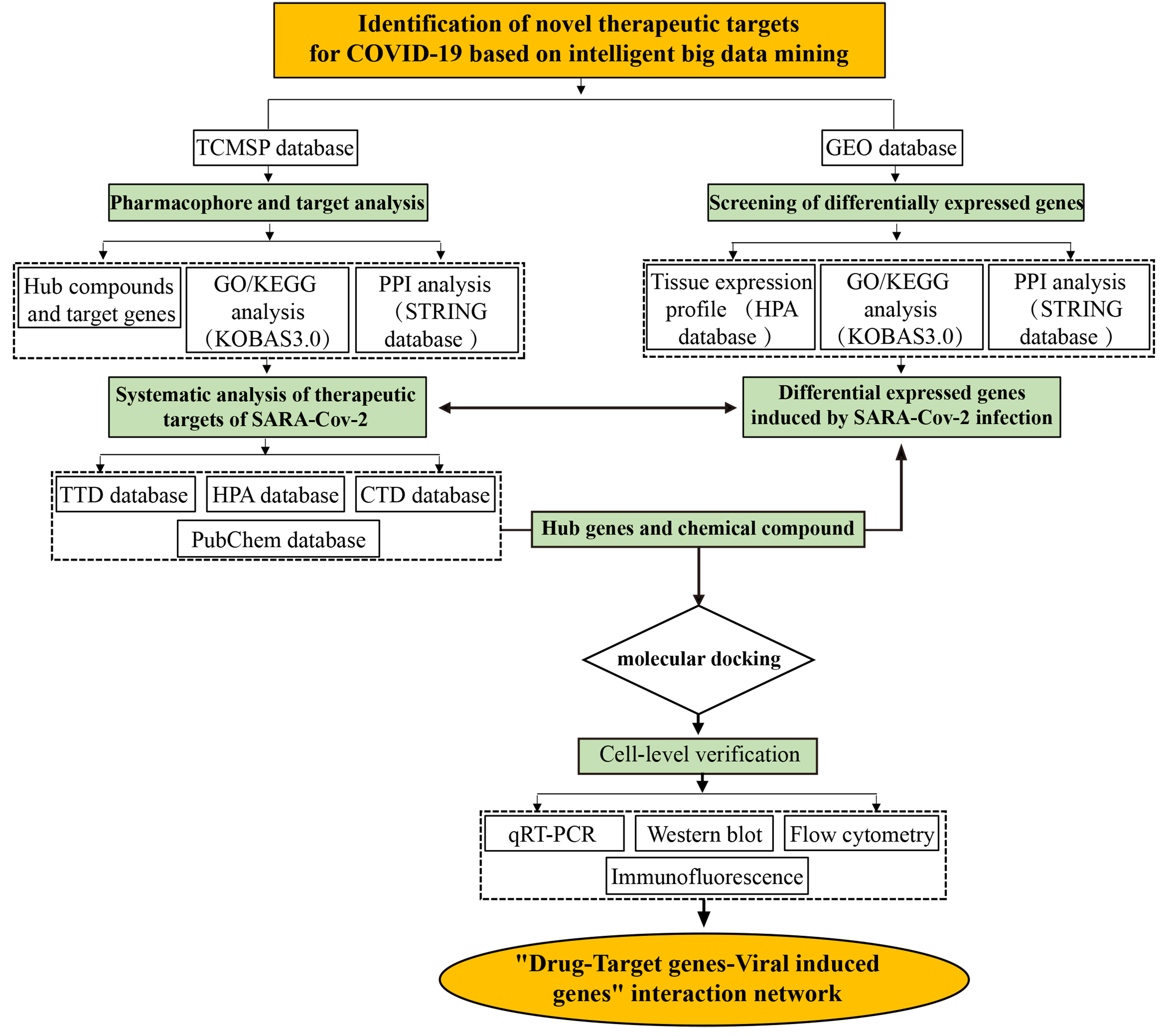

Supplement: Supplemental Material [file IPHB_A_2063341_SM5683.tif]
